# Negative feedback regulation of ABA biosynthesis in peanut (*Arachis hypogaea*): a transcription factor complex inhibits *AhNCED1* expression during water stress

**DOI:** 10.1038/srep37943

**Published:** 2016-11-28

**Authors:** Shuai Liu, Meijuan Li, Liangchen Su, Kui Ge, Limei Li, Xiaoyun Li, Xu Liu, Ling Li

**Affiliations:** 1 Guangdong Provincial Key Laboratory of Biotechnology for Plant Development, School of Life Sciences, South China Normal University, Guangzhou, China; 2 Molecular Analysis and Genetic Improvement Center, South China Botanical Garden, Chinese Academy of Science, Guangzhou 510650, China

## Abstract

Abscisic acid (ABA), a key plant stress-signaling hormone, is produced in response to drought and counteracts the effects of this stress. The accumulation of ABA is controlled by the enzyme 9-cis-epoxycarotenoid dioxygenase (NCED). In Arabidopsis, *NCED3* is regulated by a positive feedback mechanism by ABA. In this study in peanut (*Arachis hypogaea*), we demonstrate that ABA biosynthesis is also controlled by negative feedback regulation, mediated by the inhibitory effect on *AhNCED1* transcription of a protein complex between transcription factors AhNAC2 and AhAREB1. *AhNCED1* was significantly down-regulated after PEG treatment for 10 h, at which time ABA content reached a peak. A ChIP-qPCR assay confirmed AhAREB1 and AhNAC2 binding to the *AhNCED1* promoter in response to ABA. Moreover, the interaction between AhAREB1 and AhNAC2, and a transient expression assay showed that the protein complex could negatively regulate the expression of *AhNCED1*. The results also demonstrated that AhAREB1 was the key factor in *AhNCED1* feedback regulation, while AhNAC2 played a subsidiary role. ABA reduced the rate of AhAREB1 degradation and enhanced both the synthesis and degradation rate of the AhNAC2 protein. In summary, the AhAREB1/AhNAC2 protein complex functions as a negative feedback regulator of drought-induced ABA biosynthesis in peanut.

Plants encounter various environmental stresses throughout life, of which drought is one of the most serious adverse factors restricting plant survival, growth and yield. Abscisic acid (ABA), a key plant stress-signaling hormone, is produced in response to drought and counteracts the effects of this stress^1^,^2^. The accumulation of ABA under drought stress conditions is primarily due to the induction of ABA biosynthetic genes^3^,^4^. The step involving 9-cis-epoxycarotenoid dioxygenase (NCED), which cleaves xanthoxin to produce 9-cis-violaxanthin and 9-cis-neoxanthin, is thought to be rate-limiting in the ABA biosynthesis pathway^5^,^6^. In the Arabidopsis genome, there are five *NCED* (*AtNCED*) genes. Among them, *AtNCED3* plays a key role in ABA biosynthesis during water stress, when the corresponding NCED3 protein accumulates around leaf vascular tissues^7^. The *nced3* mutant line exhibits reduced ABA content, which exacerbates water stress; in contrast, overexpression of *NCED3* increases ABA content and enhances survival of water stress^3^. Genes from other species behave similarly: Zhang *et al*.^8^ transformed *MhNCED3*, the key ABA biosynthesis gene in *Malus hupehensis*, into Arabidopsis, and the resulting transgenic lines contained higher endogenous ABA levels and showed a higher drought resistance than wild-type.

The NAC (petunia NAM and Arabidopsis ATAF1, ATAF2 and CUC2) transcription factors (TFs) are plant-specific and form one of the largest TF families in plants^9^. NAC TFs function in multiple developmental processes, as well as in abiotic stress-responsive signaling. For example, NAC019, NAC055 and NAC072 are all induced by drought stress, and transgenic lines constructed with the corresponding genes show strong drought tolerance^10^. Similarly, overexpression of another NAC TF gene, *ATAF1*, results in improved drought tolerance; ChIP-qPCR (Quantitative Real-Time PCR Analysis of Chromatin Immunoprecipitation) shows that ATAF1 binding to the *NCED3* promoter correlates with increased *NCED3* expression and ABA hormone levels^11^,^12^. Several stress-inducible NAC TFs are also induced by ABA: levels of *TaNAC29*, a NAC TF from wheat, are increased by ABA treatment^13^. NAC072/RD26 regulates drought-responsive genes in the ABA-dependent pathway, as shown by upregulation of ABA-responsive genes in *RD26* overexpression lines^14^. NAC TFs often target similar consensus sequences in promoter regions, including the NACRES [NAC recognition site, CGT(G/A)] and CDBS (core DNA-binding sequence, CACG) motifs^10^. NAC019 and NAC055 can interact with the CDBS element in the promoter of *VSP1*, a jasmonic acid-induced defense response gene^15^. However, ANAC016, a NAC TF affecting drought-responsive signaling, binds to a NAC016-specific binding motif that does not contain the CDBS NAC binding motif^16^.

The ABRE binding factor/ABRE binding protein (ABF/AREB) TFs, with nine examples encoded in the *Arabidopsis* genome, have pivotal functions in ABA-dependent regulation of gene expression under drought stress and represent a subfamily of the basic leucine zipper (bZIP) TF family^17^. In *Arabidopsis, AREB1/ABF2, AREB2/ABF4* and *ABF3* are highly induced by ABA and osmotic stress treatment, while *ABF1* also controls the TFs downstream of ABA signaling, despite having a lower expression level than *ABF2, ABF3* and *ABF4*^18^. Transgenic plants overexpressing *AREB1/ABF2, AREB2/ABF4* or *ABF3* exhibit increased ABA sensitivity and improved drought tolerance^19^,^20^. The triple AREB/ABF mutant *areb1 areb2 abf3* exhibits enhanced ABA insensitivity and reduced tolerance to drought compared with wild-type^17^. Promoter analysis reveals that most ABA-responsive genes are regulated by ABREs (PyACGTGG/TC), which contain a core ACGT sequence and belong to the G-box (CACGTG) family. Various ABRE-like sequences have been reported, including coupling element (CGCGTG), CE3, *hex3* and motif III^20^,^21^,^22^.

A positive feedback mechanism regulates ABA biosynthesis in Arabidopsis, where the *ABA1, ABA2, ABA3, NCED3* and *AAO3* genes are induced by NaCl and ABA; in particular, water stress is a major factor in the induction of *NCED3*^23^. Exogenous ABA enhances the expression of *NCED3* via a distal ABA responsive element (ABRE: GGCACGTG, −2372 to −2364 bp) in its promoter^24^. However, the positive feedback regulation of *NCED3* expression by ABA raises the question of how the plant maintains ABA homeostasis during water stress. In previous work on peanut (*Arachis hypogaea*), an important cash crop, we cloned and identified *NCED1* (*AhNCED1*), *NAC2* (*AhNAC2*) and *AREB1* (*AhAREB1*)^4^,^25^,^26^. Furthermore, we showed that both *AhNAC2* and *AhAREB1* are induced by ABA in peanut, and that, in an Arabidopsis *AhAREB1*-overexpression line, transcription of *AtNCED3* is significantly suppressed^27^,^28^. The *AhNCED1* promoter was also cloned, and bioinformatics analysis showed that it contained one NACRE and four ABRE core sequence elements^29^. The ABRE element overlapped with or was located close to NACRE motifs in the −1500 to −300 bp region of the *AhNCED1* promoter. To understand the possible mechanisms of negative feedback regulation of ABA biosynthesis, we decided to investigate whether AhAREB1 and AhNAC2 can coordinate negative control of *AhNCED1* expression in peanut. In this study, we report that, during the response to ABA, AhAREB1 functions as a negative regulator of the ABA biosynthesis gene *AhNCED1*, acting synergistically with AhNAC2, with which it forms a protein complex. In addition, ABA plays an important role in water stress-induced feedback control of peanut ABA biosynthesis by affecting the stability of AhAREB1.

## Results

### AhNAC2 physically interacts with AhAREB1

**Figure 1:**
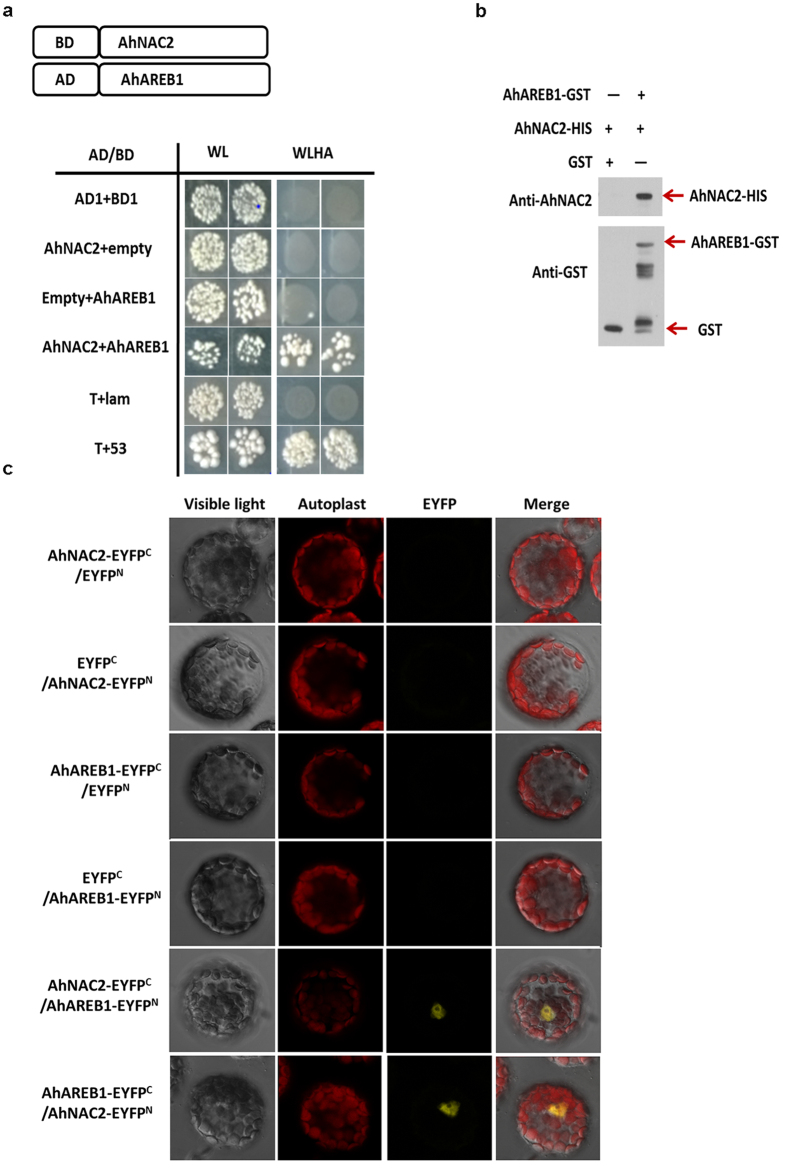
AhNAC2 physically interacts with AhAREB1. (**a**) The interaction of AhNAC2 and AhAREB1 was confirmed by yeast two-hybrid assays. Bait (AhAREB1) and prey (AhNAC2) were co-introduced by transformation into yeast strain AH109. The binding of the two TFs was confirmed by transferring the AH109 strain onto -Trp-Leu-His-Ade plus 5 mM 3-amino-1, 2, 4-triazole medium. WL: SD/-Trp/-Leu; WLHA: SD/-Trp/-Leu/-His/-Ade. (**b**) The physical interaction of AhNAC2 and AhAREB1 was confirmed by pull-down assays. Equal amounts of either GST or GST-AhAREB1 proteins were incubated with glutathione beads and then His-AhNAC2 protein was added for 4 h. After washing, samples were separated by 12% SDS-PAGE and identified by anti-AhNAC2 antibody. (**c**) AhNAC2 interacts with AhAREB1 in Arabidopsis protoplasts. AhAREB1 or AhNAC2 alone (AhNAC2-EYFP^C^/EYFP^N^, EYFP^C^/AhNAC2-EYFP^N^, AhAREB1-EYFP^C^/EYFP^N^ and EYFP^C^/AhAREB1-EYFP^N^) were used as controls.

To investigate whether AhNAC2 and AhAREB1 physically bind to each other, yeast two-hybrid assays were performed. As shown in Fig. 1a, negative control combinations, such as AD/BD, AD-AhNAC2/BD, AD/BD-AhAREB1 and T/lam, did not interact, while co-expression of AD-AhNAC2 and BD-AhAREB1 showed strong interaction, as indicated by growth on SD W-L-H-A selective plates. *In vitro* pull-down assays, using His-AhNAC2 and GST-AhAREB1 fusion proteins, were performed to verify the interaction of AhNAC2 and AhAREB1, with GST protein alone acting as a negative control. These experiments showed that His-AhNAC2 binds to GST-AhAREB1, but not GST (Fig. 1b). To further confirm the interaction between AhNAC2 and AhAREB1 *in vivo*, bimolecular fluorescence complementation (BiFC) was performed in *A. thaliana* protoplasts. A strong YFP fluorescence signal was observed when AhAREB1-EYFP^C^ and AhNAC2-EYFP^N^ or AhNAC2-EYFP^C^ and AhAREB1-EYFP^N^ were co-expressed in the same cells, while negative combinations, such as AhNAC2-EYFP^C^/EYFP^N^, EYFP^C^/AhNAC2-EYFP^N^, AhAREB1-EYFP^C^/EYFP^N^ and EYFP^C^/AhAREB1-EYFP^N^, gave only background fluorescence (Fig. 1c). In all, these results indicate that AhNAC2 and AhAREB1 form a protein complex and thus might act together to regulate target genes.

### AhNAC2 and AhAREB1 directly target the *AhNCED1* promoter region following ABA treatment

**Figure 2:**
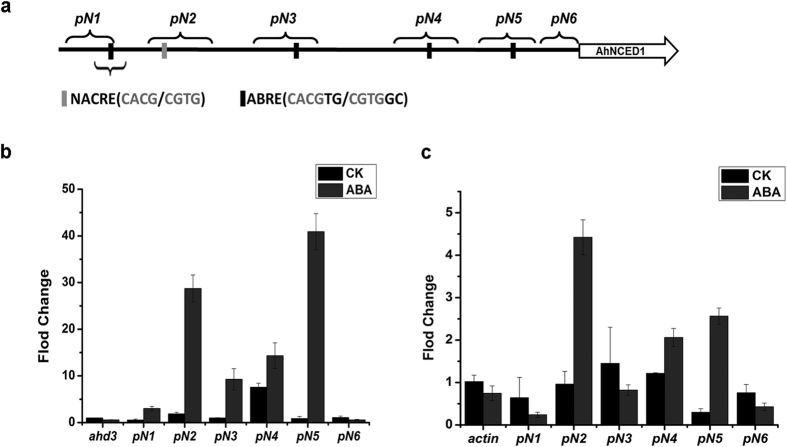
ABA promotes the binding of AhAREB1 or AhNAC2 protein with an *AhNCED1* gene promoter DNA fragment. (**a**) The positions of NACRE and ABRE elements in the *AhNCE1* promoter and the regions examined by ChIP. (**b**) and (**c**) ChIP-qPCR analysis was performed to measure the relative levels of AhAREB1 (**b**) and AhNAC2 (**c**) binding to the *AhNCED1* promoter in response to ABA (100 μM). *AHD3* was used as the control gene to measure the DNA fragment enrichment by anti-AhAREB1 antibody, while *ACTIN* was used for the anti-AhNAC2 antibody.

*AhNCED1* contains one NACRE and four ABRE core sequences in the −1500 to −300 bp region of its promoter (Fig. 2a). To examine whether AhNAC2 and AhAREB1 execute their function by binding to the *AhNCED1* promoter, a chromatin immunoprecipitation (ChIP) assay was performed in peanut. We designed five pairs (*pN1-pN6*) of primers that correspond to regions within the *AhNCED1* promoter and used PCR fragments generated from these in ChIP-qPCR assays. The results showed that, in the absence of ABA, AhAREB1 protein binding was enriched in the *pN4* region of the *AhNCED1* promoter, while AhNAC2 was not (Fig. 2b,c). However, both AhNAC2 and AhAREB1 associated with the *pN2, pN4, pN5* regions of the *AhNCED1* promoter in response to ABA (Fig. 2b,c). In the *pN1* region, which contains the most distal ABRE core sequence, only AhAREB1 was enriched in response to ABA (Fig. 2b,c). These results suggest that AhNAC2 and AhAREB1 might regulate *AhNCED1* expression by binding to specific *cis*-acting elements in its promoter in response to ABA.

### AhNAC2 and AhAREB1 co-regulate *AhNCED1* expression by feedback inhibition in response to ABA

**Figure 3:**
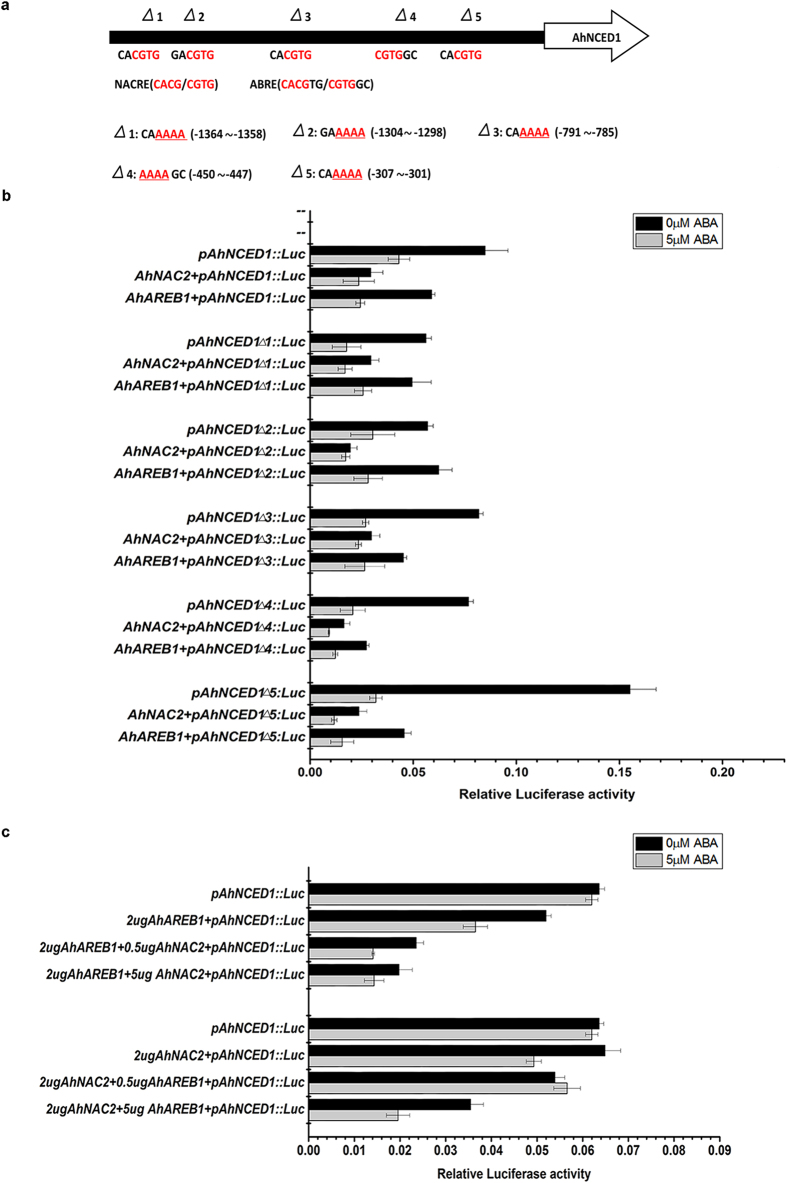
Effect of AhNAC2 or AhAREB1 on *pAhNCED1::LUC* activity during ABA treatment. (**a**) Schematic of NACRE and ABRE mutations. The triangle represents mutation of the core element. The core sequences of the NACRE and ABRE elements were mutated to AAAA. (**b**) LUC activity was measured by transient expression of *pAhNCED1::LUC* and *p35S::AhNAC2* or *p35S::AhAREB1* vectors in Arabidopsis protoplasts. A 5 μg quantity of purified vector was used per transfection. Protoplasts were incubated overnight in W5 buffer. After transfection, protoplasts were incubated overnight. (**c**) Coordinate regulation of *pAhNCED1::LUC* activity by co-expression of *AhNAC2* and *AhAREB1* with and without ABA treatment. Error bars indicate SEM (*n* = 3).

To test whether AhNAC2 and AhAREB1 regulate the expression of *AhNCED1* by binding to the ABRE or NACRE *cis*-acting elements in its promoter, we co-expressed *AhAREB1* or *AhNAC2*, and either *pAhNCED1-Luc* or *pAhNCED1Δ-Luc* (the latter has a mutation in the NACRE or ABRE motifs, where CACG/CGTG is changed to AAAA, in the *AhNCED1* promoter; Fig. 3a) vectors in wild-type Arabidopsis protoplasts. As expected, the addition of AhNAC2 or AhAREB1 significantly down-regulated the expression of *pAhNCED1-Luc*, and ABA treatment enhanced this inhibition (Fig. 3b). When *AhNAC2* and *pAhNCED1Δ-Luc* constructs were co-transfected into protoplasts, none of the individual mutations in the *AhNCED1* promoter influenced AhNAC2 inhibition of *pAhNCED1Δ-Luc* expression (Fig. 3b). However, when we transfected the *AhAREB1* construct together with *pAhNCED1Δ1-Luc* or *pAhNCED1Δ2-Luc* into protoplasts, the inhibition by AhAREB1 alone was dramatically reduced. This suggests that AhAREB1 negatively regulates *AhNCED1* transcription by binding to the ABRE element located at −1367 bp or to the NACRE motif located at −1308 bp in the *AhNCED1* promoter (Fig. 3b).

**Figure 4:**
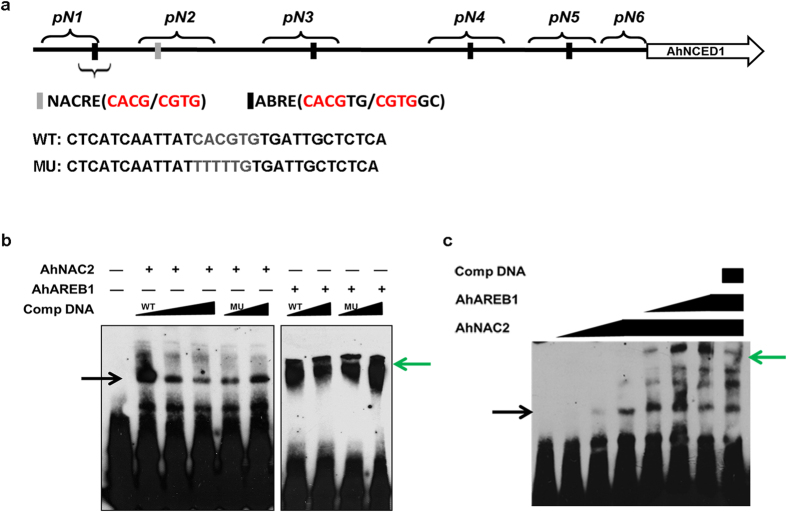
EMSA of AhNAC2 and AhAREB1 protein binding to the core NACRE element in the *AhNCED1* promoter. (**a**) Schematic of the position of the probes in the *AhNCED1* promoter. A 30 bp DNA fragment containing CACGTG was designed. WT: probe containing CACGTG; MU: probe changing CACG to AAAA. (**b**) Both AhAREB1 and AhNAC2 can bind to the same NACRE element. (**c**) Sequential addition of the two TFs into the reaction system. The black arrow indicates binding of AhNAC2 and the green arrow indicates binding of AhAREB1 to the promoter of *AhNCED1*; ‘Comp DNA’ indicates addition of competitor DNA.

To further examine whether AhNAC2 and AhAREB1 coordinately regulate *AhNCED1* transcription, *AhAREB1, AhNAC2* and *pAhNCED1-Luc* vectors were co-transfected into Arabidopsis protoplasts. We fixed the plasmid concentration of one TF, and gradually increased the concentration of the other one to identify its function in coordinate regulation. The results showed that when 2 μg *AhAREB1*, 0.5 μg *AhNAC2* and *pAhNCED1-Luc* vectors were co-transfected, *Luc* activity decreased to 37% of the control. When 2 μg *AhNAC2*, 0.5 μg *AhAREB1* and *pAhNCED1-Luc* vectors were used, *Luc* activity decreased to 84.8% of controls. Importantly, even when the amount of AhAREB1 vector transfected was increased to 5 μg, *Luc* activity was reduced to 55.8%, meaning that the negative regulation of *AhNCED1* depends on the amount of AhAREB1 protein present. In brief, the relative luciferase activities were much lower when treated with both AhNAC2 and AhAREB1 (Fig. 3c). The inhibition of *AhNCED1* promoter activity by AhNAC2 or AhAREB1 was enhanced by ABA treatment (Fig. 3c). In addition, to examine whether AhAREB1 and AhNAC2 bind directly to the core ABRE sequence, we performed electrophoretic mobility shift assays (EMSAs) using recombinant GST-AhAREB1 and His-AhNAC2 proteins and a biotinylated probe containing the ABRE cis-acting element from the −1367 locus of the *AhNCED1* promoter (Fig. 2a). A nonlabeled probe with mutated ABRE was also used as cold competitor (Fig. 2a). Both AhNAC2 and AhAREB1 were able to bind to the ABRE *cis*-acting element (Fig. 4a). Furthermore, the binding of AhNAC2 to the ABRE motif could be reduced by adding AhAREB1 protein into the reaction (Fig. 4b).

Taken together, these findings strongly support the idea that *AhNCED1* transcription is inhibited by an AhNAC2-AhAREB1 protein complex. AhAREB1 plays the key role in this negative feedback regulation, while AhNAC2 is subsidiary.

### ABA plays an important role in water stress-induced feedback control of ABA biosynthesis in the leaves of peanut

**Figure 5:**
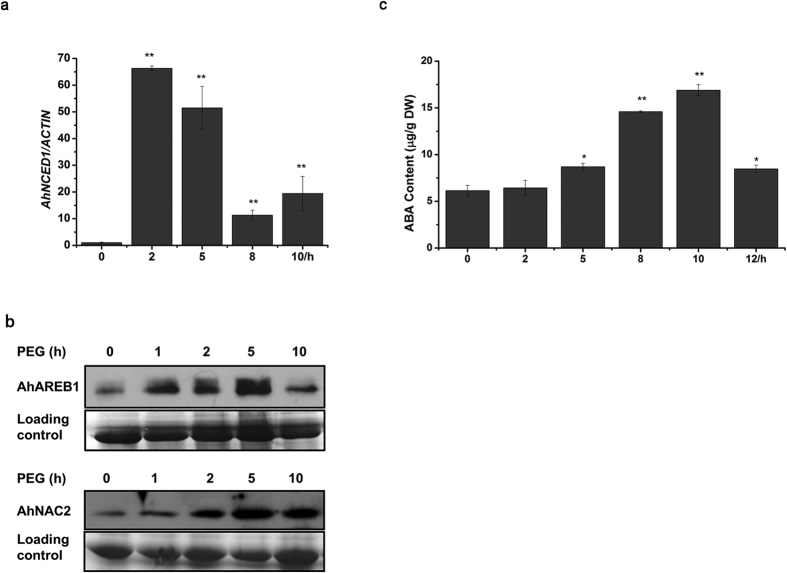
Expression of the *AhNCED1* gene, accumulation of AhAREB1 and AhNAC2 proteins, and ABA content during water stress. Peanut plants were treated with 20% PEG 6000. (**a**) Quantitative assessment of *AhNCED1* gene expression by qRT-PCR. (**b**) AhAREB1 and AhNAC2 protein levels were measured by immunoblotting using anti-AhAREB1 and anti-AhNAC2 antibodies. (**c**) The ABA content was determined by HPLC.

To investigate the relationship between the negative feedback regulation of *AhNCED1* and ABA homeostasis during water stress, quantitative RT-PCR analysis was performed. A rapid 67-fold increase in the expression of *AhNCED1* was observed after 2 h PEG treatment (Fig. 5a). However, perhaps more interesting is the subsequent significant downregulation of the *AhNCED1* gene after 8 h PEG treatment (Fig. 5a). To determine whether AhAREB1 and AhNAC2 are involved in this down regulation, immunoblotting assays were performed to assess the respective protein levels during water stress. We found that both AhAREB1 and AhNAC2 accumulated in response to PEG treatment (Fig. 5b). An HPLC assay showed that, as expected, ABA levels increased to a maximum after 10 h PEG treatment (Fig. 5c). Together with the ChIP analysis for AhAREB1 and AhNAC2 described above (Fig. 2), which showed that ABA promotes the binding of both TFs to the *AhNCED1* promoter, these results suggest that an AhAREB1/AhNAC2 protein complex is involved in the water stress-induced feedback control of *AhNCED1* expression.

### ABA influences the accumulation of both AhNAC2 and AhAREB1

**Figure 6:**
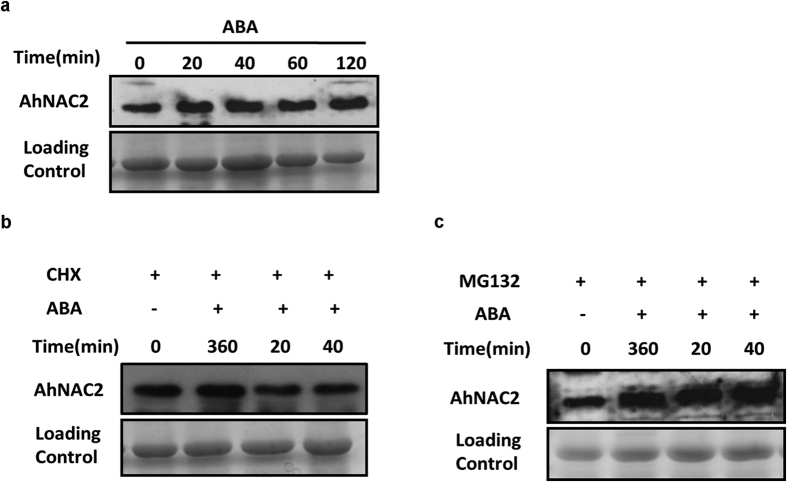
AhNAC2 protein is rapidly degraded in response to ABA in peanut. Immunoblotting of AhNAC2 protein levels in ten-day-old seedlings. (**a**) Ten-day-old seedlings were treated with 100 μM ABA and harvested at the indicated times. (**b**) and (**c**) Ten-day-old seedlings were treated with 100 μM CHX or MG132, respectively, for 6 h and then treated with 100 μM ABA and harvested at the indicated times.

To further confirm the role of ABA in this feedback regulation, we investigated the accumulation of AhAREB1 and AhNAC2 proteins in response to ABA. Both *AhNAC2* and *AhAREB1* genes are induced by ABA, but the protein expression pattern of the respective TFs is still unknown^26^,^27^. The *AhNAC2* mRNA level increased 3.5 times after 2 h ABA treatment (Supplementary Figure S1). However, the overall AhNAC2 protein levels remained stable, despite ABA treatment (Fig. 6a). Intriguingly, when peanut seedlings were pretreated with CHX (cycloheximide) for 6 h to inhibit translation, AhNAC2 protein content was rapidly reduced in the presence of ABA (Fig. 6b).

The proteasome provides the major proteolytic activity in plants, and MG132 inhibits this activity. MG132 pretreatment for 6 h stabilized AhNAC2 protein content during ABA treatment, suggesting that AhNAC2 is degraded via the proteasome (Fig. 6c). To verify this, we transferred a *p35S::AhNAC2-GFP* construct into Arabidopsis (the 35 S promoter does not respond to ABA treatment)^30^. Almost all AhNAC2-GFP protein was degraded after 3 h ABA treatment, while MG132 pretreatment prevented this (Supplementary Figure S2a). In contrast, when *p35S::AhAREB1-GFP* was transformed into Arabidopsis, AhAREB1 protein clearly accumulated in the presence of ABA, suggesting that ABA inhibits AhAREB1 breakdown (Supplementary Figure S2b).

In summary, these results demonstrate that ABA enhances both the synthesis and degradation rates of AhNAC2, but reduces the AhAREB1 degradation rate.

## Discussion

The key regulatory step in ABA biosynthesis is catalyzed by NCED, which cleaves 9-cis-epoxycarotenoids to xanthoxin^5^,^6^,^31^. In peanut, like in other plants, ABA content is markedly enhanced during water stress, in parallel with a significant increase in AhNCED1 protein levels; AhNCED1 has been identified as the key enzyme in ABA biosynthesis under water stress^32^. Previous studies demonstrated that the *NCED* gene can be induced by exogenous ABA^25^,^33^. DREB2C, ATHB and ATAF1 TFs are known to be activators of *NCED* transcription, but the negative regulation of *NCED* is still unclear^12^,^34^,^35^. However, ABA is likely to be involved, probably as part of a negative feedback mechanism, because such regulation by the product (s) of a biosynthetic pathway is ubiquitous in plants. For example, bioactive gibberellin (GA), involved in stem elongation, seed germination and root elongation, provides feedback regulation of GA 2-oxidase biosynthesis genes^36^,^37^. Here, we identified two TFs, AhAREB1 and AhNAC2, which form a protein complex in peanut to mediate ABA-dependent negative feedback regulation of *AhNCED1* transcription (Figs 1 and 3). AhAREB1 plays a central role in this process, while AhNAC2 functions as an enhancer (Fig. 3). What is noteworthy is that ABA increases the inhibitory effect of AhNAC2 or AhAREB1 on *AhNCED1* promoter activity (Fig. 3), and affects the accumulation of both AhNAC2 and AhAREB1 TFs (Fig. 5, Supplementary Figure S2). ABA achieves this by enhancing both the synthesis and degradation rate of AhNAC2 to maintain stable protein levels, while levels of AhAREB1 are increased by reducing its degradation rate (Fig. 5, Supplementary Figure S2).

The relationship between NAC and AREB TFs, which has mostly been researched in *Arabidopsis*, is complex. The expression of three *Arabidopsis* NAC TFs, ANAC072, ANAC019 and ANAC055, is induced in response to ABA, and a yeast one-hybrid assay revealed that key AREBs (ABF3, ABF4) bind to the promoters of all three of the corresponding genes via the ABRE core *cis*-acting element, which occurs several times within each NAC promoter^10^,^38^. *NAC016* is also involved in drought stress responses: overexpression of *NAC016* results in low drought tolerance, while *nac016* mutants have high drought tolerance, suggesting that NAC016 works as a negative regulatory factor in drought stress. Furthermore, both NAC016 and the product of its target gene *NAC-like, activated by AP3/PI (NAP)* directly bind to the promoter of *AREB1* and repress its transcription^16^. AREB1 encodes a TF with a central role in the stress-responsive ABA signaling pathway.

A previous study suggested that ABA affects ABF1 and ABF3 protein accumulation by post-translational regulation, while ABA can suppress the interaction between the ubiquitin E3 ligase KEEP ON GOING (KEG) and ABFs to slow their proteolysis^39^,^40^. Levels of NAC1, an *Arabidopsis* NAC TF with a role in the auxin signaling pathway, can be increased by treatment with MG132, indicating that NAC1 may be regulated by a post-translational mechanism^41^. Our results are consistent with the conclusion that AhAREB1 is the key negative regulator of *AhNCED1* expression, while AhNAC2 is simply an enhancer of this inhibitory effect. Furthermore, both AhNAC2 and AhAREB1 can improve the drought tolerance of the plant by inducing stress-related gene expression^27^,^28^, meaning that both TFs are activators of the ABA signaling pathway.

The negative feedback regulation of ABA biosynthesis in peanut, mediated by AhAREB1, contrasts with the situation in Arabidopsis, where a positive feedback mechanism of ABA biosynthesis regulation has been reported, with the *cis*-acting element ABRE playing a critical role^24^. In tomato, similarly to peanut, a negative feedback mechanism has been found^42^. Thus, water stress causes a rapid increase in endogenous ABA in WT tomato leaves, such that high levels are attained after 6 h, which continue to increase through until 24 h of the treatment. At the same time, the expression of *NCED1* reaches a maximum at 6 h after imposition of the stress and then reduces until the 24 h time point. Similar observations were made in tomato root^42^. Bioinformatics analysis of the 2000-bp upstream region of *NCED1* (GenBank Accession no. SGN-U577478) in tomato showed that it contains two *cis*-acting ABRE elements and three *cis*-acting NACRE core elements (Supplementary Figure S3). This might indicate that the negative feedback regulatory mechanism of ABA biosynthesis we observed in peanut is a more general phenomenon in plants, even though it has not yet been found in Arabidopsis. The regulation of ABA biosynthesis is likely to be complex, however, involving both positive and negative mechanisms. In this regard, we note that, although AhAREB1 acts on the *AhNCED1* promoter as a negative transcription factor in response to ABA treatment (Fig. 2), only the *cis*-acting elements in the *pN1* and *pN2* regions are involved in this negative regulation. We predict that *cis*-acting elements in other regions of the *AhNCED1* promoter might feature in other forms of regulation of *AhNCED1* transcription.

**Figure 7:**
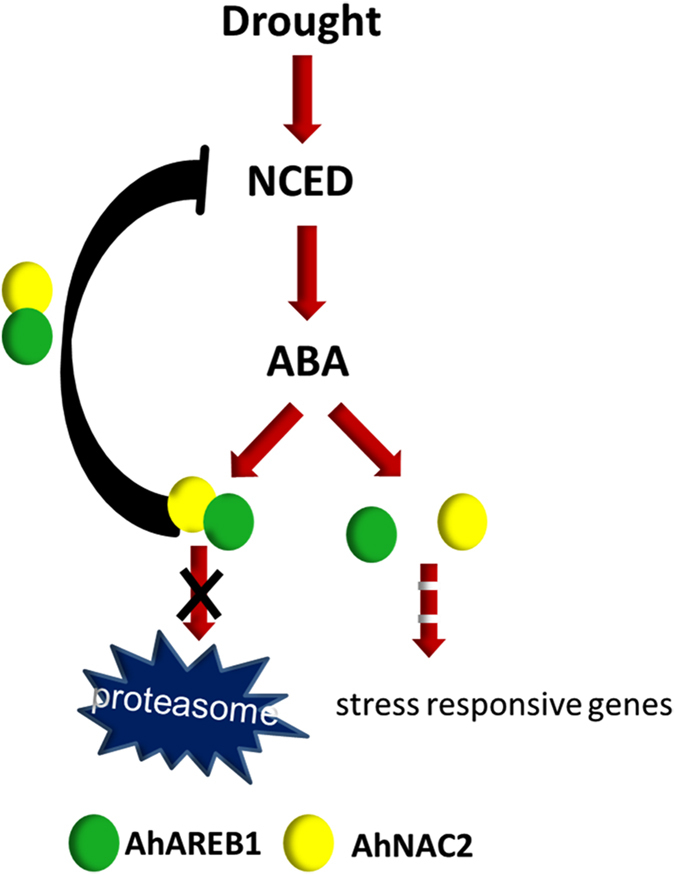
Model of the dual role of the AhAREB1/AhNAC2 protein complex in peanut. Drought induces AhNCED1 expression and ABA levels increase. Levels of the AhNAC2/AhAREB1 protein complex also increase as both AhNAC2 and AhAREB1 accumulate. The protein complex binds to the *AhNCED1* promoter to partially inhibit its transcription and maintain ABA homeostasis. The two TFs also activate the ABA signal pathway by upregulating downstream genes.

The results described in this paper are all consistent with the following description of events. After 2 h PEG treatment in peanut, the expression level of the *AhNCED1* gene reaches a peak. The level of AhNAC2/AhAREB1 protein complex increases as both AhNAC2 and AhAREB1 proteins accumulate, leading to partial inhibition of *AhNCED1* expression. ABA improves both the synthesis and degradation rate of AhNAC2 protein and slows AhAREB1 protein degradation so that the latter accumulates within cells. AhAREB1 plays the key role in the feedback regulation of *AhNCED1* transcription, while AhNAC2 is an enhancer of this process. Both AhNAC2 and AhAREB1 are activators in the response to water stress and induce the expression of stress-responsive genes (Fig. 7).

## Materials and Methods

### Plant growth and treatments

Seeds of peanut (*Arachis hypogaea*) were sown and grown as described^43^. Yueyou 7 is a line provided by the Crop Research Institute, Guangdong Academy of Agricultural Sciences, China. PEG 6000 (w/v) was used to simulate the effect of drought stress^43^. For ABA, cycloheximide (CHX) or MG132 treatments, four-leaf-stage (10–12 days after sowing) plants were carefully removed from the soil mixture and then grown hydroponically. ABA, CHX and MG132 were applied by uniformly spraying onto leaf surfaces at a concentration of 100 μM^39^. Peanut leaf samples (100 mg) were frozen in liquid nitrogen immediately following the treatments and stored at −70 °C until further use.

### Yeast two-hybrid assays

Yeast two-hybrid (Y2H) assays were performed as previously described^43^. AhAREB1 and AhNAC2 cDNAs were transferred into the plasmids pGADT7 and pGBKT7, respectively. Yeast AH109 was cotransformed with special-purpose vectors (pGADT7, pGBKT7, pGADT7-AhNAC2/AhAREB1, pGBKT7-AhNAC2/AhAREB1). To avoid self-activation, yeast was treated with 5 mM 3-amino-1, 2, 4-triazole (3-AT; Wako) for Y2H screening.

### Pull-down assays

Full-length *AhAREB1* and *AhNAC2* cDNAs were cloned into pGEX-4T-1 (Pharmacia) and pPROEX-HTa vectors to allow production of GST-AhAREB1 and His-AhNAC2 fusion proteins after induction by isopropyl-D-1-thiogalactopyranoside (IPTG). Expression of GST-AhAREB1 was induced in *Escherichia coli* BL21-Codon Plus-RP (Agilent Technologies) by adding IPTG to a final concentration of 0.5 mM at 30 °C for 4 h, after which the bacteria were transferred to 25 °C overnight. His-AhNAC2 was expressed in *E. coli* BL21 (DE3) (Amersham Biosciences) by adding 0.5 mM IPTG at 37 °C for 4 h. GST pull-down assays were performed as described^41^. Glutathione Sepharose beads (Amersham Biosciences) or Ni-NTA agarose beads (Millipore) were used to purify fusion proteins. Two μg His-AhNAC2 protein was incubated with immobilized GST or GST-AhAREB1 in binding buffer (50 mM Tris-HCl pH 8.0, 100 mM NaCl and 1 mM EDTA) at 4 °C overnight. Proteins retained on beads were separated by SDS-PAGE and detected with anti-GST (Millipore) or anti-His antibody (Sigma), as appropriate, by immunoblotting (see below).

### BiFC experiments

For BiFC experiment assays, full-length *AhAREB1* and *AhNAC2* cDNAs were cloned into the pGreen binary vector HY105 containing C- or N-terminal fusions of EYFP to generate *35 S:AhAREB1-EYFP*^*C*^ and *35 S:AhNAC2-EYFP*^*N*^, respectively, which were then cotransformed into Arabidopsis protoplasts as previously described^4^. AhNAC2-EYFP^C^/EYFP^N^, EYFP^C^/AhNAC2-EYFP^N^, AhAREB1-EYFP^C^/EYFP^N^ and EYFP^C^/AhAREB1-EYFP^N^ worked as negative control. YFP fluorescence signals were observed after 12–20 h incubation under a fluorescence microscope (Leica). All figures show representative images from three independent experiments.

### Antibody preparation, protein extraction and immunoblotting

The AhAREB1 coding sequence was cloned in the pPROEX-HTa vector and the resulting fusion protein His-AhAREB1 was induced under the same conditions as GST-AhAREB1. His-AhAREB1 and His-AhNAC2 proteins were purified and used for antibody production (polyclonal, rabbit). Antibody specificity was tested using the respective purified recombinant protein and also total protein from peanut leaves. Protein was extracted from peanut leaves (100 mg) by grinding leaves in liquid nitrogen and 1 mL lysis buffer (50 mM Tris-HCl pH 7.2, 10% glycerol, 2% SDS, 1% β-mercaptoethanol, protease inhibitors cocktail (Roche), 100 mM PMSF (Sigma)). Anti-GFP (abcam, ab290) was also used to test the accumulation of AhAREB1-GFP and AhNAC2-GFP fusion proteins in transgenic Arabidopsis.

### Chromatin coimmunoprecipitation (ChIP) assay

For the ChIP assay, leaves of four-leaf-stage (10–12 days after planting) peanuts (500 mg) were fixed with cold MC buffer (10 mM potassium phosphate pH 7.0, 50 mM NaCl, 0.1 M sucrose, 1% formaldehyde) for 20 min by vacuum concentration. Nucleoproteins were isolated by the method published by Su^42^ and sonicated to produce DNA fragments on the order of 300 bp. Five μg anti-AhNAC2, anti-AhAREB1 and rabbit IgG (Millipore) were used for immunoprecipitation and antibody complexes were recovered by Protein G PLUS-Agarose (Santa Cruz Biotechnology). Specifically precipitated DNA was recovered and analyzed by real-time PCR with SYBR Premix ExTaq Mix (Takara Bio). The peanut genes *ACTIN* and *AHD3* (Genbank: DQ873525.1 and EE127230.1, respectively) were used to calculate the relative fold-enrichment of target DNA fragments. The primers used to measure the binding of the TFs to the *AhNCED1* promoter are listed in Supplemental Table 1.

### Quantitative Real-Time PCR (qRT-PCR)

RNA was extracted as described by Wan and Li^25^. Reverse transcription was carried out using PrimeScript™ RT reagent Kit with gDNA Eraser (Perfect Real Time) (Takara). SYBR^®^ *Premix Ex Taq*™ (Tli RNaseH Plus) (Takara) was used according to manufacturer’s instructions with an ABI PRISM 7300 Sequence Detection System (Applied Biosystems, UK). Primers for qRT-PCR are listed in Supplemental Table 1.

### Electrophoretic mobility shift assay (EMSA)

Purified His-AhNAC2 and GST-AhAREB1 recombinant proteins were used for protein-DNA binding. The EMSA assay was performed using the LightShift Chemiluminescent EMSA kit (Pierce). A 30 bp DNA fragment containing CACGTG in the upstream of P1 region in the *AhNCED1* promoter was used as probe. Nonlabeled probe contain the native core-sequence (CACGTG) or mutated core-sequence (TTTTTG) was used as cold competitor.

### LUC complementation assay

The *AhNCED1* promoter was amplified and cloned into the pGreenII 0800-LUC vector, while *AhNAC2* and *AhAREB1* cDNAs were cloned into pGreenII 62-SK, the effector vector in the LUC complementation assay. LUC luminescence of live protoplasts was measured as previously described; 5 mM ABA was added^44^,^45^.

### Determination of ABA content

Leaves (0.5 g) were collected, frozen in liquid nitrogen and then ground with 8 ml methanol:glacial acetic acid (80:20), as described by Yue^46^. High performance liquid chromatography (HPLC) was used, with an ABA standard (Sigma) diluted to 10 mM, 1 mM, 100 nM and 10 nM as needed. ABA contents were measured in triplicate for each sample.

## Additional Information

**How to cite this article**: Liu, S. *et al*. Negative feedback regulation of ABA biosynthesis in peanut (*Arachis hypogaea*): a transcription factor complex inhibits *AhNCED1* expression during water stress. *Sci. Rep.* **6**, 37943; doi: 10.1038/srep37943 (2016).

**Publisher's note:** Springer Nature remains neutral with regard to jurisdictional claims in published maps and institutional affiliations.

## Supplementary Material

Supplementary Information
