# Supplementary material for: Negative feedback regulation of ABA biosynthesis in peanut (Arachis hypogaea): a transcription factor complex inhibits AhNCED1 expression during water stress
Source: Sci Rep. 2016 Nov 28;6:37943. doi: 10.1038/srep37943 (PMC5124963; doi:10.1038/srep37943)
Supplement: Supplementary Information [file srep37943-s1.pdf]

Negative feedback regulation of ABA biosynthesis in peanut (*Arachis hypogaea*): a transcription factor complex inhibits *AhNCED1* expression during water stress

Shuai Liu[1], Meijuan Li[1], Liangchen Su[1], Kui Ge[1], Limei Li[1], Xiaoyun Li[1], Xu Liu[2], Ling Li[1,*]

[1] Guangdong Provincial Key Laboratory of Biotechnology for Plant Development, School of Life Sciences, South China Normal University, Guangzhou, China.

[2] Molecular Analysis and Genetic Improvement Center, South China Botanical Garden, Chinese Academy of Science, Guangzhou 510650, China. Correspondence and requests for materials should be addressed to L. L. (email: liling502@126.com)

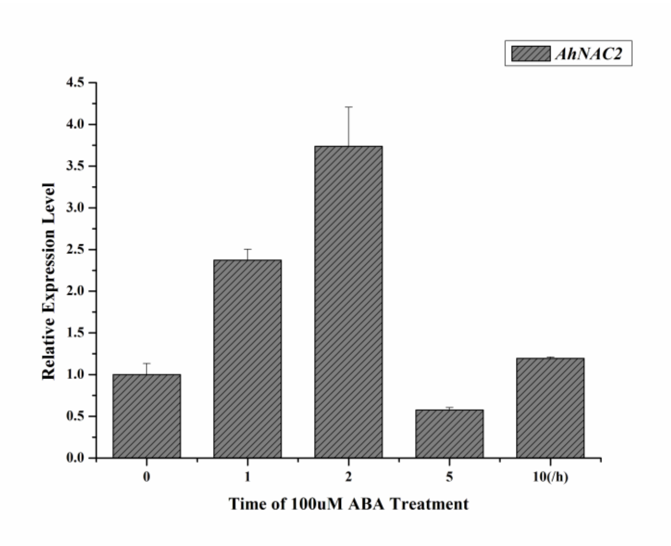

Supplementary Figure S1 Expression of *AhNAC2* in response to ABA.

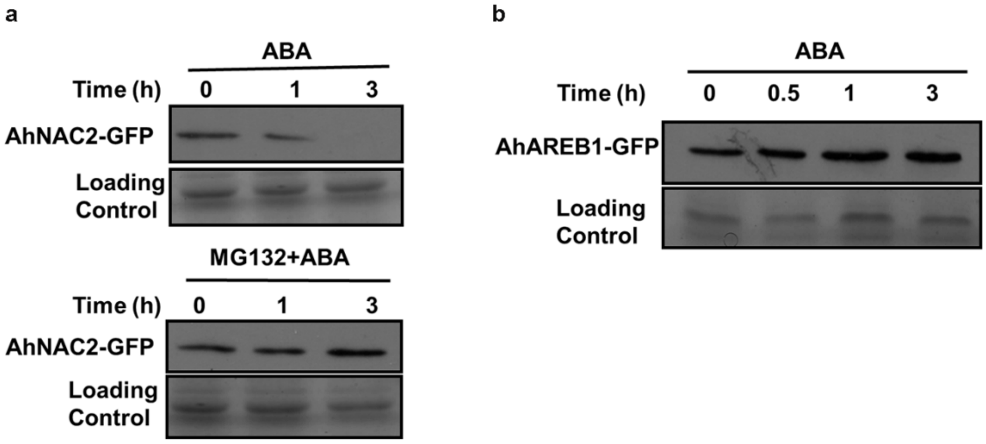

Supplementary Figure S2 AhNAC2 or AhAREB1 protein accumulation is affected by ABA treatment in corresponding over-AhNAC2-GFP or over-AhAREB1-GFP transgenic *A. thaliana* lines. (a) The effect of ABA on AhNAC2-GFP protein levels with or without MG132 pretreatment in a *p35S::AhNAC2-GFP A. thaliana* line. (b) The effect of ABA on AhNAC2-GFP protein levels in a *p35S::AhAREB1-GFP A. thaliana* line.

TTCAAAAAGGATGACATTATTTGACTTGAAATGGAGTTTAAGAAAAAGAAAAAAGATTTTTTAATTTTATAATTCTAAATTAAAGTTGTG
TCAAATATACCAAAATGCGCTTTAATTTTATGATCTTAAATATGTCACGTGGCAAGTTAAAGTTAAAATGTTACAAAAAAATAAATGAATA
ATTCTTTTTGAAACAGACTTAAAAGGAAACATGGTCATTCTTTTTTAAATGGAAGGAATATTTAAATTTCTAAATTAAAAAACATCCATCT
ACGATTAAATGTAATTGTAATAAAATAAAATGTTTCTACAATTTTTTTAAGAAAGAATTATGTTATAATTTCTTCTTTCTTAATTTATGTAATC
TTTTTATTGAAAATTTTTCAAAAGGATAACATTTTTAAATTTAGTATAAATTATAATTTAACCTTTTTTTTTAAAAATATATGTAAGTAATATT
ATGACATAAATTGAAATTAGAGGAGTAGATTATTTACGCCTTCAACATGAGCGCGTGAGGAAGTAGGCAAAGAAACACTTTTATAGTTG
CTGCACATTTTATGAAACAATAAAAAGTGCGCATTCAATAGTACTGCTCACTCCTGACTACTGCTTTGGCGAGTTGTTAACATGTTTTTTT
TTATTAGGATGGTGCACCATAAAATTTATTTTTTTTCATATTTAAGATTTGAATTATGATTTTTTCAGTATTCAAATTTTAAACTTTTAGTTAA
AGCACGAGTGGAAATCAATTTACCTTTTTAGTTATATGTTAAACATTTAATTAATTGTATAATTTATTTGTTTTTTTTATATAAGAAGCTAGC
AAATAACAATAGATAAATTTTAAAAATATTATACCTAAAAATTTTGATTTATTTCTTATTCAATTCGTCCTTATATACATTTTCTCAGTTCAAA
TAGAATCGTCCACAAGAACTTCGCATTTTCTTTAAACACATATAATCCTTTAAATTTGCTCTTATTTTTTATTTTCATAATCTATCTTAGTCTT
GTTTCATTTTGATCCTCATGCTTCTTTTTCAAATAAAGCTAAGGGGTTAAAATGAAATAAGACTAAGCTAGAGTGTCAATATGAAAAACG
AGGGCATGTTAACATGACTATCTATGTATTTGGTCTCTCTTTTATTTTATTTATCTAATTTTAACTTGACATGCAAATAACACTAATAAAAAT
AAATTTAGTGAAATTTTAAAAATTTTAAATTTGAAAAGGATAAAATATATCCGATGTTATCACTATATCTCCTAGGTAGATATAGATTGTTTT
AAAAAAATTATCAATCCAAATAAATTAATGCAGTGATAAAAAAAAATATTACGAATAATAGCAAAGTAGTGCATAGCATATATGCAAGAGAT
CATATACTTGGAATATCAAACTACATTATATAAAATGAGATGAAGGGAGTATCAACTTTTTTTTGTATTTATTTAAAAGAAATGTATTGAGA
ACACTCCTAAACTACGCATGAATTATTAGTTTCATTCTTGAATTATTGACAGAACTAAAGACACTTATTTAGTTGACTAACTAGACTAAAAT
ACACCATAATTTTGTAATATGAGCGAAACACCTCAAAATTTCTCATTAAATTTTAGGAATACTTTCAATACTTTTCTCATTTTTATTAATAAT
CTCATTCTCCTACAAAAGTTTACGCTCATTTGTTATGTCGTGTTGCATATCAATACTTGTCGTTGGTTTATTTAATTGAATTTTCGATTTATG
AGTTTGACATTAATACCCCTACCCATTATGTTGCTCCCAAGTGACCTTCTCACAAATACCACCACGTGCTCTTCTCTTAGATGTCTAAGGT
GGACCTTCGTTCCCCTCTTCACCATACACACTCCCTTTTTTTCCTCAAACATCTTCTTCTATTTAAACCCTCTTCTTTCCCTCTCTCTTCTAC
TCAACCCCAACAATTCTCTTTTCTCCCTTTTTTTTTTCCTCTCTTCAAAGAAGGAAAAAAAAAT

Supplementary Figure S3 The bioinformatics analysis of the 2000-bp upstream region of NCED1 in tomato. The yellow highlights indicates ABRE *cis*-acting elements and green indicates *cis*-acting core NACRE elements.

Supplemental Table 1 Primers used in the paper

Name	Sequence
Actin QF	GATTGGAATGGAAGCTGCTG
Actin QR	CGGTCAGCAATACCAGGGAA
adh3F QR	GACGCTTGGCGAGATCAACA
adh3F QR	AACCGGACAACCACCACATG
pN1-F	GCAAGCGTGTGGGGTAGTAA
pN1-R	GAGTGAGAGTTGAGAGTGTC
pN2-F	CAACAAACAAACCAAAATGC
pN2-R	TAGGAGGGGTGTGAAGAAGA
pN3-F	TAATAAAGCAAAAGAAACTCAATTTA
pN3-R	TGAGAAAGTTGAGGGATGAGGGGAG
pN4-F	CCTTTCCTTTCTCTTGTTATTTCCA
pN4-R	TGATACACTGCCACGAACACTCTAC
pN5-F	TCTTGATCACTTATTGGCAATCCAACT
pN5-R	TTGATTGAAGAATAGTAATGGGTGGT
pN6-F	ACTCACAAACCACCTCTC
pN6-R	GCAAGTAAGTAGCAGGGG
AhNCED1 QF	TTACCTGTGGGATTGTTTGC
AhNCED1 QR	ACATGAGCCTCTACTTCTGC

Full-length gels and blots

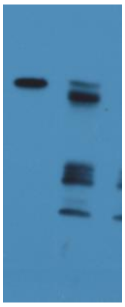

Figure 2b Anti-GST

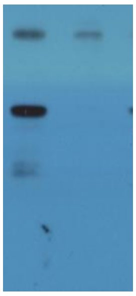

Figure 2b Anti-AhNAC2

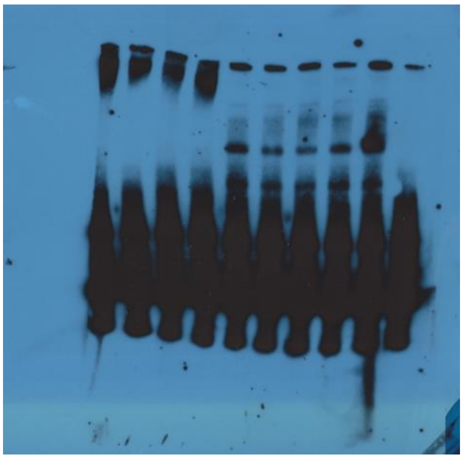

Figure 4b

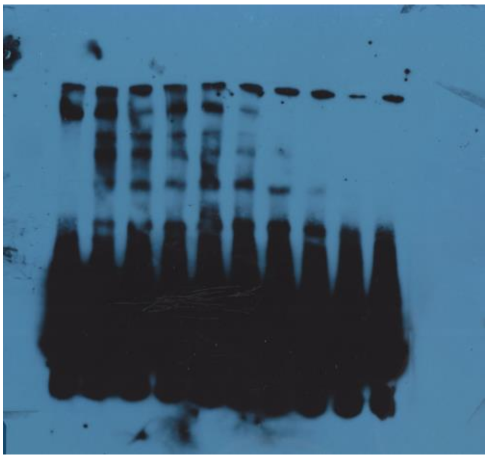

Figure 4c

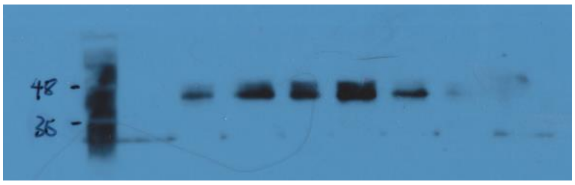

Figure 5b AhAREB1

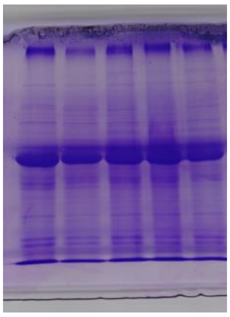

Figure 5b AhAREB1 loading Control

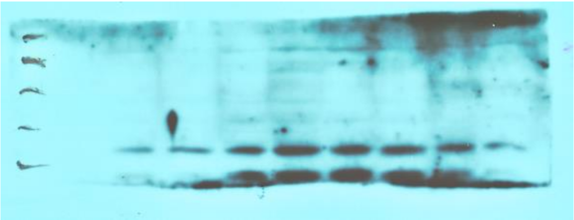

Figure 5b AhNAC2

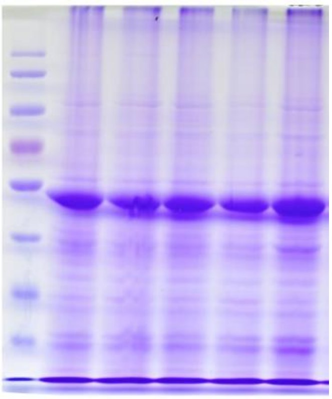

Figure 5b AhNAC2 loading Control

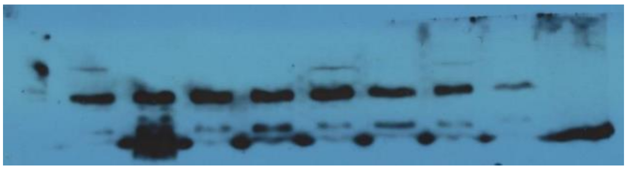

Figure 6a AhNAC2

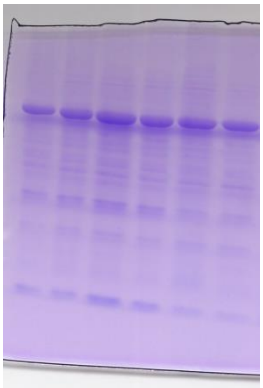

Figure 6a AhNAC2 loading Control

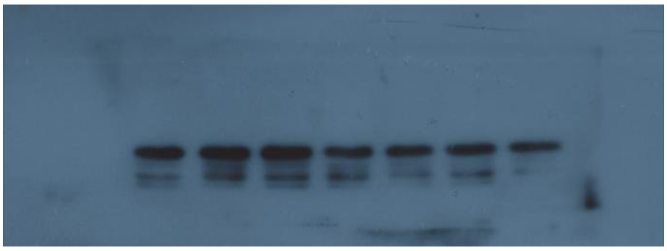

Figure 6b AhNAC2

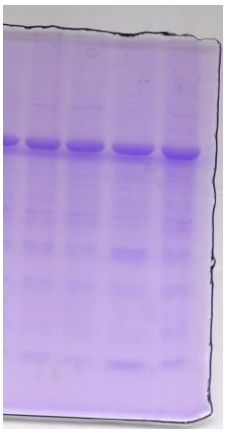

Figure 6b AhNAC2 loading Control

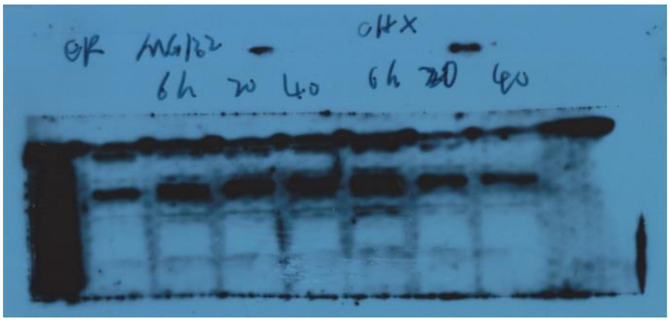

Figure 6c AhNAC2

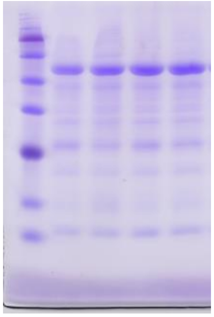

Figure 6c AhNAC2 loading Control

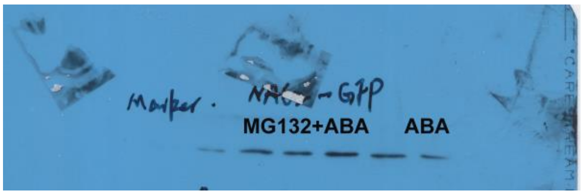

Supplementary Figure S2a

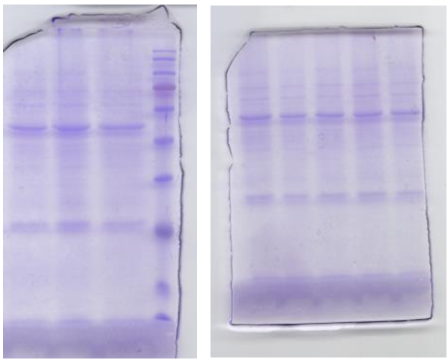

Supplementary Figure S2a loading control

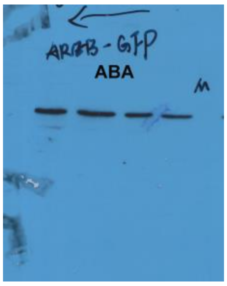

Supplementary Figure S2b

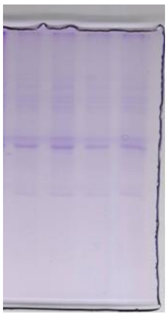

Supplementary Figure S2a loading control
